# Effects of HepaSphere microsphere encapsule epirubicin with a new loading method transarterial chemoembolization: in vitro and in vivo experiments

**DOI:** 10.1007/s12672-023-00831-y

**Published:** 2023-11-22

**Authors:** Wen Zhang, Nan Du, Liangwen Wang, Jiaze Yu, Minjie Yang, Wei Zhang, Xvdong Qu, Jianjun Luo, Zhiping Yan

**Affiliations:** 1 Department of Interventional Radiology, Zhongshan Hospital, Fudan University, No. 180 Fenglin Road, Xuhui District, Shanghai, 200032 China; 2 Shanghai Institute of Medical Imaging, Shanghai, 200041 China; 3 National Clinical Research Center for Interventional Medicine, Shanghai, 200032 China

**Keywords:** Transarterial chemoembolization, Pharmacokinetics, Drug-eluting beads, Epirubicin

## Abstract

**Methods:**

HS microspheres were loaded in a solution of hypertonic saline and contrast medium at different ratios. Morphology, size distribution, and drug loading capacity of the microsphere were evaluated. Rabbits with hepatic VX2 tumors underwent conventional TACE, drug-eluting beads TACE with HS microsphere loading epirubicin by recommended method (dTACE) or a new loading method (ndTACE). The plasma and tissue epirubicin concentration, tumor necrosis, and the microsphere distribution within the tumor were assessed.

**Results:**

It was found that the mean diameter of HS microspheres was effectively reduced to 102 ± 14 μm after loading with 10.0% NaCl and Ultravist (370 mg I /mL) at a ratio of 2: 8 ml. The loading capacity reached 78.7%. It was noted that the concentration of tumor epirubicin was significantly higher (p = 0.016) in the ndTACE group (11,989.8 ± 5776.6 ng/g) than the concentration in the dTACE (6516.5 ± 3682.3 ng/g) and in cTACE groups (1564.1 ± 696.1 ng/g, p < 0.001). Further, the tumor necrosis in group with the new loading method (ndTACE) was 92.4%.

**Conclusions:**

The size of HS microsphere can be effectively reduced when it is loaded with a mixture of hypertonic saline and non-ionic contrast material. HS microsphere loaded with epirubicin using the new method (ndTACE) can increase the drug concentration in tumor and hence exert better improved antitumor effect.

## Key points

- The diameter of HepaSphere microspheres can be reduced effectively by using a new loading method.
- In vivo study on the rabbit VX2 liver cancer model suggests that the new loading method can increase anticancer drug concentration in tumor and hence exert better effect.

## Introduction

In the past 3 decades, transarterial chemoembolization has been the mainstay of treatment for unresectable hepatocellular carcinoma (HCC) [1, 2]. Conventional transarterial chemoembolization (cTACE) was typically performed by injection of chemotherapeutic agents with lipiodol emulsions and subsequent embolic particles such as gelatin sponge, polyvinyl alcohol or calibrated microspheres [3]. The limitation of this technique is well known for arterial obstruction could be heterogeneous and as time lapse, a large proportion of the chemotherapeutic drug released to the systemic circulation. This resultantly limits the dose of the chemotherapeutic agent used and hence restrict the therapeutic effect of cTACE.

Transarterial chemoembolization using drug-eluting beads (DEB) loaded with chemotherapy drug have demonstrated better plasma pharmacokinetic profile and higher concentration of chemotherapy drugs in the tumor compared with cTACE [4]. Moreover, drug-eluting beads transarterial chemoembolization (dTACE) does not have the obvious systemic effects and provides other advantages including better patient tolerability and sustained, time-released delivery of chemotherapy into the tumor. However, existed studies fails to demonstrate superiority of dTACE over cTACE in terms of overall survival [5, 6].

Transarterial chemoembolization efficacy depends on the combination of cytotoxicity and the embolic effect. Previous studies have shown that the size of drug-eluting embolic material used are large than 300 μm, whereas diameters of 100–300 μm is used only in a few centers. According to Namur et al*.* [7], TACE with DEB of between 100 and 300 μm blocks vessels with a mean diameter of 237 μm, which distribute both inside and in the periphery of the tumor. It was reported that the mean distance which DEB penetrated inside the HCC nodule was 3.8 mm from tumor boundary. Therefore, the diameter of bead is important in determining the distribution of the DEB inside or outside of tumor. Further, the diameter of bead determines the amount of drug that is delivered to the lesion or to the surrounding liver parenchyma.

Recent prospective and retrospective studies have showed that smaller DC Bead M1 (between 70 and 150 μm) has an objective response rate ranging from 77 to 93% [8–10]. Elsewhere, Embozene TANDEM 75 μm microspheres (Boston Scientific) loaded with 150 mg of doxorubicin (DOX) provided a high local tumor control (95%) in a small cohort of patients who mainly had unilobar disease [11]. Another prospective study has reported that 45 patients treated with Hepasphere (HS) microsphere (between 30 and 60 μm) showed an objective response rate of 68.9% with no serious adverse effects [5].

HepaSphere (Merit Medical) is a biocompatible, nonabsorbable, expandable and loadable microsphere [12]. It swells to approximately four times their diameter in 0.9% NaCl aqueous solution and non-ionic contrast media compared with their initial dry diameter. In comparison with DC Beads (100–300 μm), the current smallest size specifications of HS microsphere (30–60 μm, in dry state), can expand to between 166 and 242 μm in saline and between 145 and 213 μm after loading with DOX [13]. In vitro studies have demonstrated that HS microsphere diameters expands in human serum [14]. The slightly larger expansion of microsphere in human serum match with morphology of the vessel lumen. This leaves no space between the arterial wall and the particle itself and hence provides a permanent occlusion and delivery of the chemotherapeutic drug locally [15, 16].

In a more recent study, Takeshi’s et al*.* [17] reported that the expanding level of HS microspheres can be reduced by using hypertonic saline. They performed their studies using a mixture of non-ionic contrast material and hypertonic saline for drug loading. It was found that the mean diameter of cisplatin-loaded HS microspheres (50–100 μm in dry size) was reduced to 188.4um compared with the control expansion (404.9 μm).

The current study performed an in vitro and in vivo experiments to explore a new loading method towards reducing the expansion of HS microspheres (30–60 μm in dry state).

## Materials and methods

### In vitro study

Vials (25 mg) of commercially available HS microsphere (Merit Medical), 30–60 μm in dry state, were used for all sample series. Clinical grade epirubicin Hydrochloride for injection (Pfizer) and nonionic Ultravist (370 mg I /mL) (Bayer) were used in this study.

Various solvent media composed of different ratio of normal saline (N/S, 0.9% NaCl), hypertonic saline (N/S, 10.0% NaCl) and nonionic contrast Ultravist (370 mg I /mL) were prepared. Commercially available epirubicin (30–50 mg) was then added to these solvent media. The subsequent solutions were then exposed to vials of HS microsphere. Each vial of HS microspheres was agitated briefly and allowed to incubate for 2 h at room temperature. After completion of drug-loading, the mean diameter and the size distribution were evaluated using a digital microscope (Leica, GER). Supernatant (about 100 uL) was collected, from each sample and analyzed using a validated high-performance liquid chromatography (HPLC) method to quantify the loading efficacy of HS microsphere. Each method was repeated three times. The method which effectively reduced the diameter of microsphere after loading drugs was selected as a new loading method for further study.

### In vivo study

This study was approved by the Committee on Animal Affairs in Zhongshan Hospital, Fudan University. All experiments in this study were performed according to the animal care requirement of The ARRIVE guidelines. All methods were performed in accordance with the relevant guidelines and regulations.

Adult New Zealand white rabbits (weighting between 2.5 and 3.0 kg) were used for this study. The VX2 tumors were transplanted in the hind limb of a carrier rabbit. When the tumor size reached about 1 cm in diameter the tissue was harvested and cut into small cubes (approximately 1mm^3^) for subsequent tests. The tumor pieces were implanted surgically into the left lobe of liver of each rabbit. The liver tumors with a diameter of 1.0 cm (2 to 3 weeks after implantation) were considered as an ideal condition for embolization. Animals with a liver tumor over 2 cm in diameter, severe malignancy, multiple liver metastases, or weight loss of more than 20% were excluded from the experiments of this study. Forty rabbits with VX2 liver tumors were divided into four groups: the control group (n = 8), the conventional TACE (cTACE) group (n = 8), the recommend drug-loading method TACE (dTACE) group (n = 12) and the expansion reduction drug-loading method TACE (ndTACE) group (n = 12).

Nothing was performed until euthanasia in the control group. 4 mg epirubicin dissolved in 0.2 ml saline, and reconstituted with 0.4 ml lipiodol to constitute an epirubicin emulsion. The epirubicin emulsion mixed with contrast medium was used for hepatic artery chemoembolization in the cTACE group. For the dTACE group, 20 ml of epirubicin solution at a concentration of 2.5 mg/mL was used for drug-loading in a vial of HS microsphere. After loading completion, 8% of one vial microsphere (4 mg epirubicin) was used for embolization in each animal. For the ndTACE group, HS microspheres were prepared according to the results of the part of the in vitro study, and each animal was embolized with the HS microsphere solution containing 4 mg of epirubicin. For TACE group, the planned final dose of epirubicin for each animal was 4 mg. The epirubicin emulsion injection was terminated in advance, when reflux to gastric vessel occurred and the amount of epirubicin injected was recorded.

### Procedure of transarterial chemoembolization

All procedures were performed by a certified interventional radiology physician. Right femoral artery was exposed, separated by surgical incision and a 4-F introducer sheath (Merit Medical) was introduced. A 4-F RH catheter was introduced and then a 1.9-F microcatheter (Merit Medical) was used to perform selective angiography of the celiac trunk. A branch of the left hepatic artery was super selectively catheterized and the tumor feeding artery were also reconfirmed by artery angiography. The treatment was performed in the different groups, taking care to avoid reflux.

### Pharmacokinetics study

To measure serum epirubicin concentrations, 2 ml of blood was obtained before and at 10, 30, 60 and 180 min after TACE. Each sample was centrifuged at 3,000 rpm for 10 min at 4 ℃ and the plasma was immediately frozen using liquid nitrogen and stored at -80 ℃. All the serum samples were processed for HPLC analysis.

Necropsy and liver harvest were performed in all groups, 7 days after embolization. Tumor and adjacent hepatic parenchyma were immediately weighed, frozen using liquid nitrogen and stored at -80 ℃ for epirubicin concentration measurements, the remaining tumor pieces were fixed in 10% buffered formalin and stained with hematoxylin and eosin (H&E) to assess tumor necrosis. The rates of tumor necrosis were calculated as a percentage of the tumor necrosis area by an independent pathologist blinded to the treatments. Each slide (through the largest dimension of tumor) was marked in the software and converted into a percentage. And the percentage of the tumor necrotic area in the entire tumor area was calculated in by using the Image J software.

Epirubicin concentration was analyzed according to a previously published protocol [18, 19]. This was done through liquid chromatography-tandem mass spectrometry (HPLC–MS/MS) as well as using a TSQ Quantum Ultra mass spectrometric detector (Thermo Fisher Scientific) and a Dionex UltiMate 3000 RSLC Nano (Thermo Fisher Scientific). To measure epirubicin concentration in the serum, blank serum samples (190 uL) were spiked with epirubicin standard solution (Shanghai Kewel Chemical Technology Co.) (10 uL) to make the standard curve in serum, ranging from 5 ng/mL to 5000 ng/mL. The lowest limit of quantification for epirubicin was set as 5 ng/mL. The plasmatic samples were pretreated with methanol for protein precipitation. A column (2.1 × 100 mm, 1.9 μm; Thermo Hypersil GOLD) was used and the flow rate was set as 0.5 ml/min. The injection volume of standards or samples was 5 μL.

Epirubicin concentration in the tumor regions and adjacent hepatic parenchyma were measured by using HPLC method as mentioned earlier. According to a previously published protocol [20], each tissue was mixed with PBS (1:5, v/v) and then homogenized. The subsequent pretreatment method was the same as that for plasma.

### Statistics analysis

Statistical analyses were performed using SPSS Statistics software version 24.0 (SPSS Inc.). The plasma and tissue drug concentration data were analyzed using a one-way analysis of variance model (ANOVA), whereas multiple comparisons were determined by LSD test. The level of significance was set at *p* value < 0.05.

## Results

### The diameter and loading efficiency of HS microsphere

**Fig. 1:**
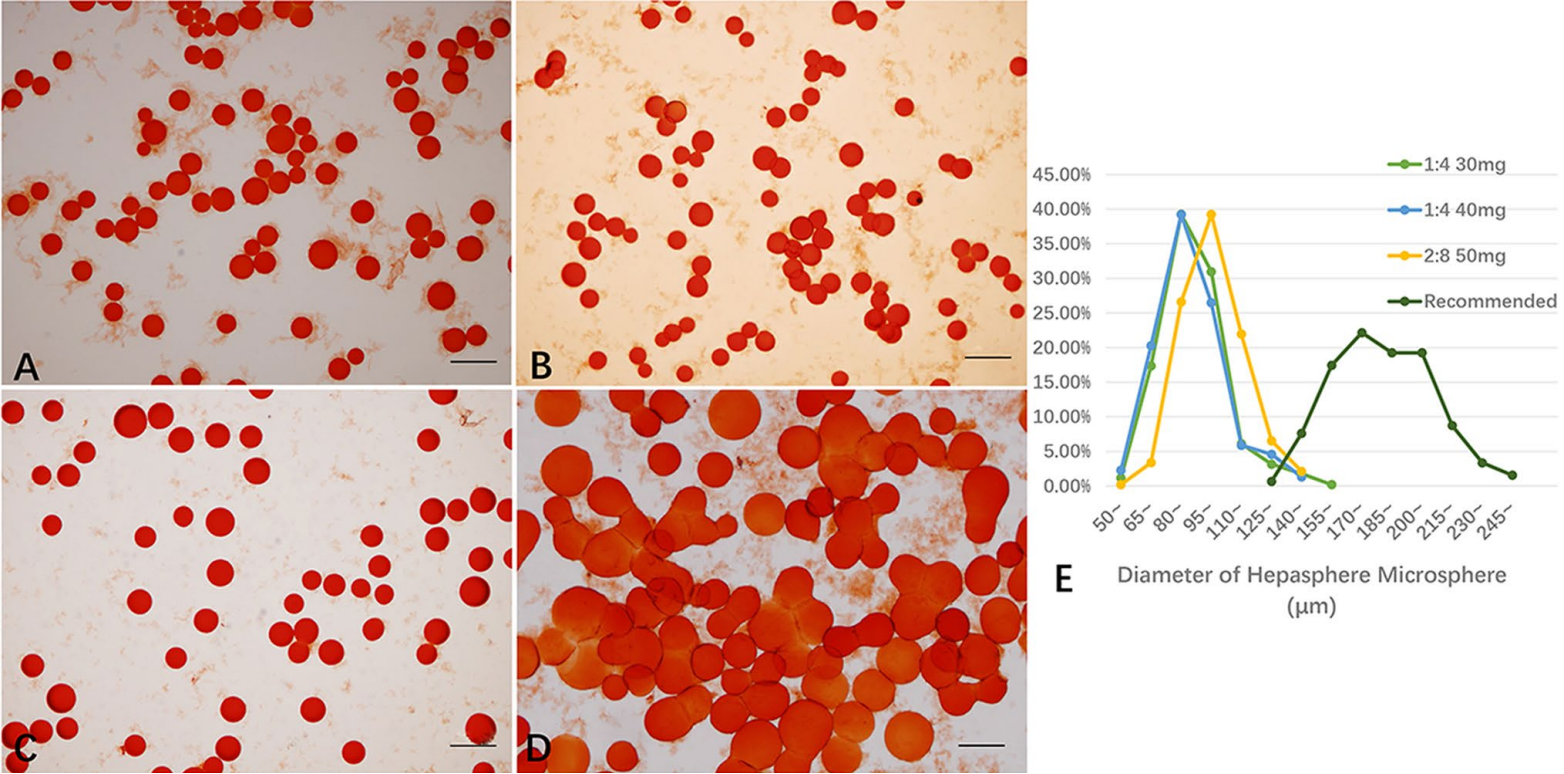
Microscopic images of epirubicin-loaded HS microsphere. The morphology of HS microsphere loaded 30 or 40 mg epirubicin in emulsion composed of 10% NaCl 1 ml and nonionic contrast 4 ml (**A**, **B**). HS microsphere loaded 50 mg epirubicin in emulsion composed of 10% NaCl 2 ml and nonionic contrast 8 ml (**C**). HS microsphere loaded 50 epirubicin with normal saline (**D**). Cumulative size distributions of HS microsphere by different loading method (**E**). Scale bar “_” 200 μm (> 200 expended HS per sample)

It was found that the mean diameter of HS microsphere was between 93 ± 13 μm and 102 ± 14 μm, after loading with solvent media composed by hypertonic saline (N/S, 10.0% NaCl) and nonionic contrast Ultravist (370 mg I /mL) at a ratio of 1 ml: 4 ml and 2 ml: 8 ml, respectively. The mean diameter was 185 ± 28 μm for HS microsphere after loading by recommended method. The loading efficiency of epirubicin with hypertonic saline and contrast medium system was between 90.2% ± 0.7% and 78.7% ± 3.6% when prepared at a ratio of 1 ml: 4 ml or 2 ml: 8 ml, respectively (Table 1). Size distributions of HS after loading through different methods were shown as in Fig. 1. To avoid insufficient occlusion of the tumor microarteries or reflux of dense microsphere for premature embolization, the mixture using 2 ml of 10% NaCl and 8 ml of non-ionic contrast material (Ultravist 370 mg I /mL) loading 50 mg epirubicin was determined as the new loading method for the animal study.

**Table 1:** Hepasphere size distribution and drug-loading efficiency by different methods

10% NaCl (ml)	0.9% NaCl (ml)	Ultravist 370 (ml)	Epirubicin (mg)	Diameter (um)	Diameter range (um)	Drug-loading efficacy (%)
–	20	–	50	185 ± 28	124–251	99.5 ± 0.1
1	–	4	30	93 ± 13	57–141	90.2 ± 0.7
1	–	4	40	93 ± 13	62–135	89.5 ± 1.1
2	–	8	40	103 ± 14	65–153	83.2 ± 1.6
2	–	8	50	102 ± 14	63–151	78.7 ± 3.6
2	2	8	50	128 ± 16	85–167	–
4	2	8	50	109 ± 14	63–162	–

### Transarterial chemoembolization procedures with HS microsphere

For the groups which received HS microsphere injection, the mean duration of time taken for microsphere injection was > 10 min for reaching the intended amount of microsphere injection and avoiding vessel spasm. For the dTACE group, 10 out of the 12 rabbits reached the intended amount of microsphere injection. The retrograde flow to the gastrointestinal arteries was observed in the other 2 rabbits, and the injection was prematurely terminated. In the ndTACE group, the total amount of HS microsphere suspension was delivered in 11 out of 12 rabbits, excepting 1 rabbit with severe vasospasm caused by hepatic artery injury. Three rabbits in dTACE group died whereas two rabbits died in the ndTACE group.

### Plasma and tissue pharmacokinetics

**Fig. 2:**
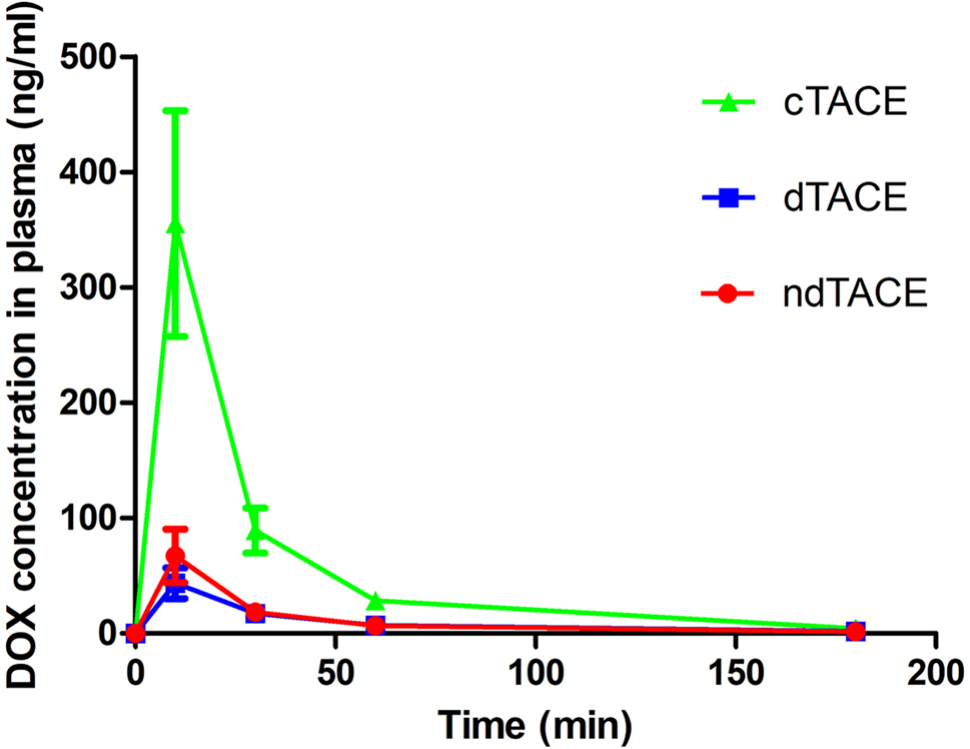
Plasma epirubicin concentrations curves after TACE in different groups

The plasma epirubicin concentration peaked at 10 min in all groups and the returned to near baseline at approximately 60 min and keep a low level until 180 min after TACE (Fig. 2**)**. The peak plasma levels of epirubicin were 355.2 ng/ml, 43.6 ng/ml and 67.2 ng/ml in the cTACE group, dTACE group, and ndTACE group, respectively **(**Table 2**)**. The peak plasma epirubicin concentration were significantly lower in dTACE and ndTACE group than those in cTACE group (*p* = 0.001 and *p* = 0.034). And no statistical difference was found between the ndTACE group and the dTACE group (p = 0.138). The AUC at 0–180 min for total plasma epirubicin concentrations of the ndTACE group and dTACE group was 2088 ng/mL•min and 1733 ng/ml•min, respectively, while that of the control group was 9947 ng/mL•min.

**Table 2:** Epirubicin concentration (ng/g in tissues) in tumor and adjacent hepatic parenchyma region 7 days after intervention

Region	cTACE group	dTACE group	ndTACE group
Plasma C_max_ (ng/ml)	355.2 ± 97.8*	43.6 ± 13.3	67.2 ± 23.2
Plasma AUC (ng*min/ml)	9947	1733	2088
Tumor (ng/g)	1564.1 ± 696.1*	6516.5 ± 3682.3	11,989.8 ± 5776.7
Adjacent hepatic parenchyma (ng/g)	87.5 ± 53.6*	746.9 ± 451.0	490.3 ± 403.8

^*^p < 0.05 compared to dTACE group

Data present as mean ± SD

Post hoc: LSD

**Fig. 3:**
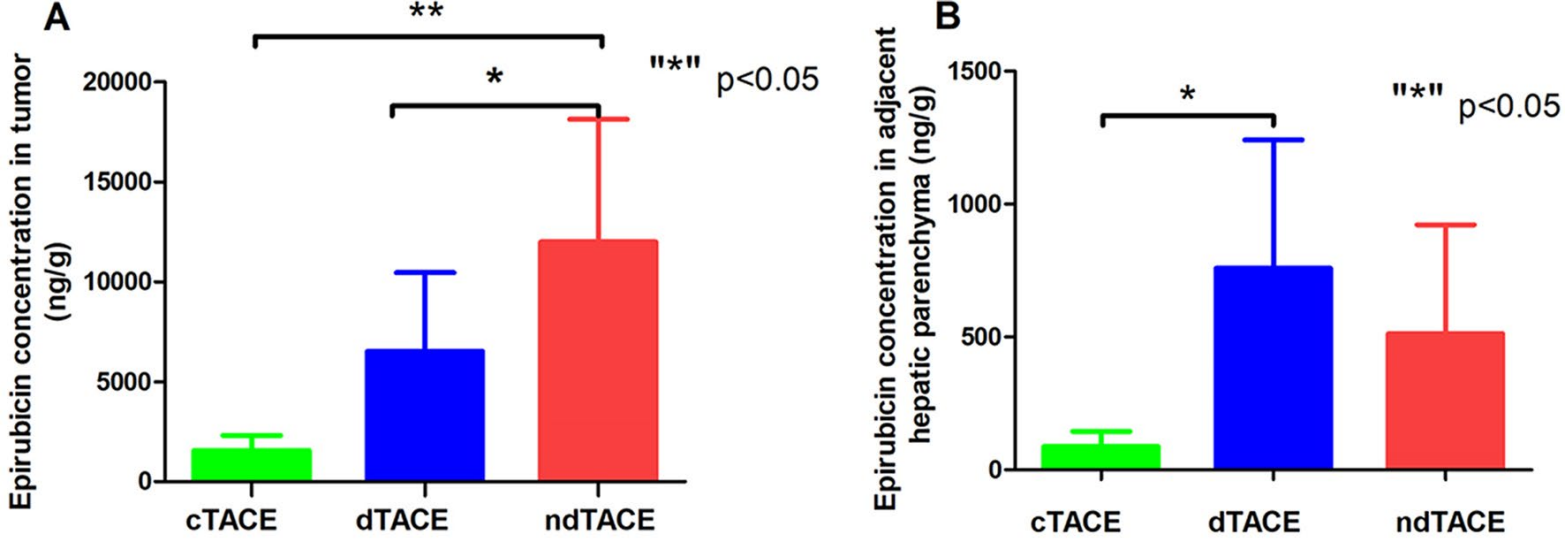
Epirubicin concentrations in tumor tissue **A** and in adjacent hepatic parenchyma **B** 7-days after TACE (“*” p < 0.05)

The mean epirubicin concentration in tumor tissue in ndTACE group (11,989.8 ± 5776.6 ng/g) were significantly higher than those of the dTACE group (6516.5 ± 3682.3 ng/g, *p* = 0.016) and those of the cTACE group (1564.1 ± 696.1 ng/g, *p* < 0.001). The adjacent hepatic parenchyma epirubicin concentration values in the dTACE (758.5 ± 452.2 ng/g) group were significantly higher than those in the cTACE group (87.5 ± 53.6, *p* = 0.027), and no significant difference were found between the dTACE group and the ndTACE group (512.5 ± 385.8, *p* = 0.184) **(**Fig. 3**)**.

### Histologic features of HS microspheres

**Fig. 4:**
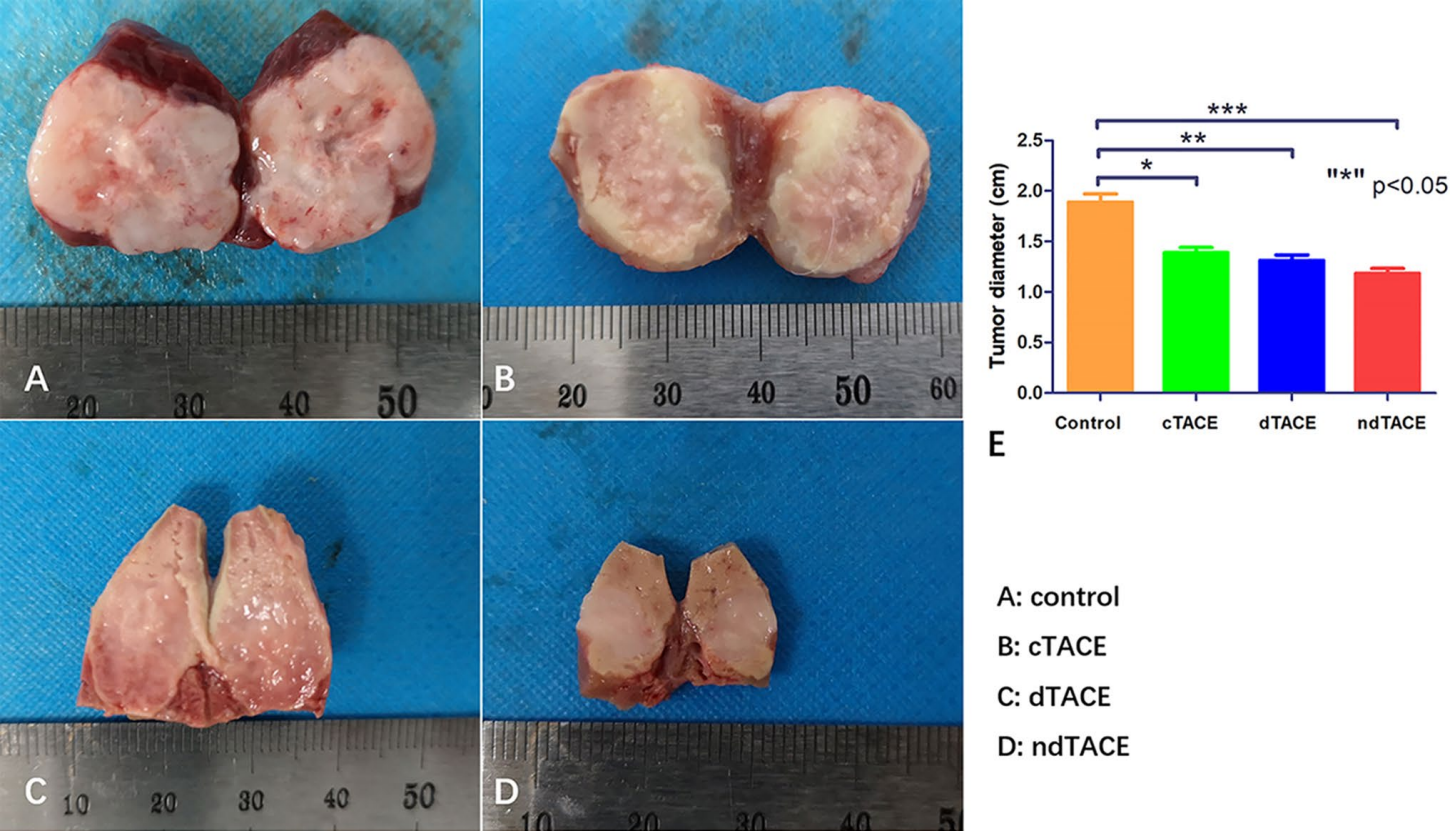
Representative tumor specimen in the control (**A**), cTACE (**B**), dTACE (**C**), and ndTACE (**D**) groups 7-days after treatment. Tumor diameter comparation in each group (**E**) (“*” p < 0.05)

On histopathology, tumor diameter in the control group, cTACE group, dTACE group, and ndTACE group were 1.9 ± 0.22, 1.4 ± 0.14, 1.3 ± 0.15 and 1.2 ± 0.14, respectively **(**Fig. 4**)**. On the other hand, the tumor diameter in control group was obviously larger than those in other three treatment groups and no significant statistical difference were found in three groups receiving TACE treatment.

**Fig. 5:**
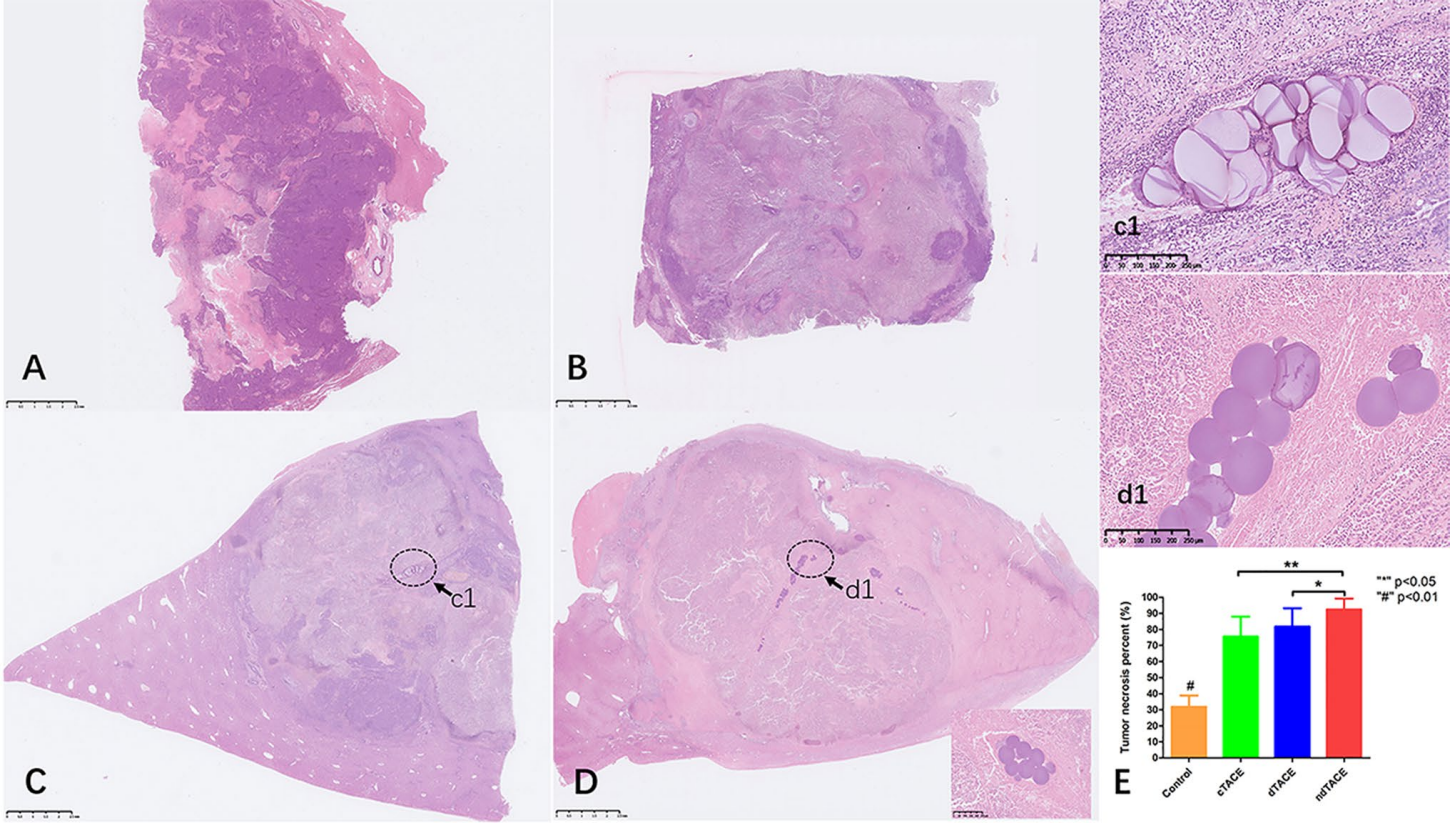
Pathology images of H&E-stained slides of tumours in control (**A**), Ctace (**B**), dTACE (**C**) and ndTACE (**D**) group. c1 and d1, local magnification of the elliptical region in Figures **C** and **D**, showed the microsphere morphology within the tumor vasculature. Tumor necrosis percent comparation in each group (**E**) (“*” p < 0.05, “#” p < 0.001)

The mean tumor necrosis percent was 31.9% ± 6.6% in the control group, 75.5% ± 11.6% in the cTACE group, 81.6% ± 10.7% in the dTACE group, and 92.4% ± 6.4% in the ndTACE group **(**Fig. 5**)**. Among the three treatment groups, the ndTACE group showed significantly higher tumor necrosis rates compared with the dTACE group (*p* = 0.030) and the cTACE group (*p* = 0.001). Without any treatment, the percent of spontaneous tumor necrosis rates in the control group were significantly lower (*p* = 0.001) than those receiving TACE treatment.

Both single and cluster HS microsphere were identified inside the tumor and adjacent parenchyma. However, a greater number of microspheres, especially in single HS microspheres, were found in the ndTACE group. Compared to the recommended method, the ability of the microspheres to swell and deform is limited by the new loading method.

## Discussion

According to the Barcelona Clinic Liver Cancer (BCLC) classification, transarterial chemoembolization is the gold standard therapy for patients at intermediate stage. These are specifically the patients presenting with large or multinodular HCC with preserved liver function [3, 21]. Transarterial chemoembolization is divided into the cTACE and dTACE depending on different drug delivery system. Conventional TACE typically performed using the antitumor drug mixed with lipiodol injection and reinforced the embolism with particle material. It also varies in nature and stability hence fails to show a controlled release pattern [22]. However, since introduction, the past one decade, the drug-eluting beads, ranging from 100 to 700 μm, are the most common specifications used in clinical cases and experimental studies [23, 24]. Smaller sized microspheres, such as TANDEM (40 μm) and DC-Beads (between 70 and 150 μm), were developed to reach into smaller tumor feeding vessels hence showing a superior efficacy and increased necrosis of the target tissue [25, 26].

Although, HS microsphere, typically swells to approximately four times their dry diameter in 0.9% NaCl aqueous solution and non-ionic contrast media, it has been proven that the expanding level can be reduced by using hypertonic saline [17]. Further, epirubicin shows less toxicity than DOX while having the same anticancer activity [27]. Therefore, the current in vitro study was conducted in rabbits with VX2 liver cancer to confirm the feasibility and efficacy of HS microsphere (between 30 and 60 μm) using a new loading method. This in vitro study showed that HS microspheres (between 30 and 60 μm), which expanded to about 60–150 μm, can be reduced by using hypertonic saline of 10% NaCl and Ultravist (370 mg I /mL) at the ratio of 1:4. According to the recommend loading method, results of this study showed that HS microsphere (30–60 μm in dry state) expanded to around 185 μm, which confirmed to the information provided by the manufacturer. In line with previous study [17], it was found that the diameters hardly changed when HS was loaded at a fixed proportion of hypertonic saline and contrast medium at different epirubicin concentrations. This effective expansion reduction of HS microsphere by the new loading method further explains that HS microsphere swollen and deformable ability may determine by the concentration of sodium chloride ions in the solution. Furthermore, the loading efficiency of HS microspheres through the new loading method could reach about 80%-90%.

The in vivo study showed that the ndTACE and the dTACE groups exhibited low C_max_ of plasma concentration of epirubicin, which was significantly higher in the cTACE group. The results of this study were consistent with the findings of a previous study where DOX loaded DC beads exhibited lower C_max_ compared with cTACE by different lipiodol emulsions [20]. A similar plasma concentration of epirubicin in the ndTACE group and the dTACE groups can possibly explain the drug loading efficiency and stable sustained drug release property of HS microsphere prepared by the new loading method. The concentration of epirubicin in tumor and adjacent liver parenchyma was lower in the cTACE group compared with the drug concentration in the TACE groups using HS microsphere. These results were in agreement with previous studies, conducted by Hong et al*.* [23] or Gupta et al*.* [28].

It is worth mentioning that the current study, the tumor epirubicin concentration in the ndTACE group was statistically higher than the concentration in the dTACE group. This could be due to the increased number of HS microsphere retention in ndTACE group tumor. Furthermore, the results of HS microsphere retention in tumors of the two groups in the present study is in consonance with the findings of a previously published study by Gholamrezanezhad et al*.* [26]. Gholamrezanezhad et al*.* reported that more beads and higher tissue doxorubicin levels were observed in the studied group of DC Beads having diameters between 70 and 150 μm than in the group with Beads having diameters between 100 and 300 μm. Consistent to the findings of several previous studies, the HS microsphere in the dTACE group of current study deformed to conform to the morphology of the vessel lumen and adjacent particles [15, 28]. However, the deformation of HS microsphere in ndTACE group was not through visual assess. Therefore, it is concluded that the difference may have been attributed by the fact that smaller beads size in the ndTACE group limited the degree of deformation. Hence, it remains need more study to test some mechanical characterization of the HS microspheres loaded by the conventional versus new method.

Results of this study showed that the mean rate of spontaneous necrosis was 32% in 3 to 4 weeks after implantation. This was consistent with the findings of a previous study which conformed that VX2 tumors are prone to partial baseline necrosis [29]. This was potentially because the low vascular density cannot provide enough nutrition for rapid growing tumors. The tumors also showed necrosis in all groups which received TACE compared with control group in this study. However, it was evident that there was no significant difference of tumor necrosis percentage between the cTACE and dTACE groups. This could be due to an increased the embolization efficacy of lipiodol in cTACE, since the cTACE could not only block the tumor micro-vessels but also diffuse into the tumor parenchyma [30]. It was noted that the enough size of HS microsphere in dTACE group sufficiently restricted it penetrating into tumor. The ndTACE group had significantly higher tumor necrosis percentage compared with the dTACE and cTACE groups at 7 days after treatment. This showed that high-dose epirubicin can be delivered into the tumor for abundant HS microsphere deposition. This result was in accordance with previously published findings which shows that a higher number of small DC Beads (between 70 and 150 μm) reaches the tumor core, accompanied by higher doxorubicin levels in tumor tissue compared with the larger DEBs (between 100 and 300 μm) [26].

The current study has some limitations. First, only HS microspheres with a dry size of 30–60 μm were used in the study. Therefore, to clarify effectiveness of the new loading method, there is need for investigation using recently launched HS microspheres (between 20 and 40 um in dry state), which are currently the smallest available products. Second, the concentration of epirubicin in each group was only evaluated within 180 min after TACE. This could not verify the longtime drug releasing results of HS microsphere by new loading method. Finally, the experiment did not include liver enzymes measurement results that reflects hepatic parenchymal damage degree in each group. Furthermore, to guarantee the equal dose of epirubicin injection, the HS microsphere solution retrograde to the non-target liver parenchyma was higher in the dTACE than in ndTACE group. Therefore, the liver enzyme difference was not compared.

In conclusion, it was evident that the expansion of HS microspheres can be effectively reduced after loading in fluid composed of a mixture of 10% NaCl and non-ionic contrast material, especially at the ratio of 1: 4. Furthermore, the HS microsphere loaded with the new method showed low plasma epirubicin concentration and adequate drug loading capacity which was similar to the recommended loading method. Therefore, the current in vivo study on the rabbit VX2 liver cancer model suggests that the new loading method can increase anticancer drug concentration in tumor and hence exert better effect.

## Data Availability

The data used to support the findings of this study are available from the corresponding author upon appropriate request.

## Abbreviations

TACE: Transarterial chemoembolization
cTACE: Conventional TACE
dTACE: Drug loading beads TACE
ndTACE: Drug loading beads with new loading method TACE
DEB: Drug-eluting beads
HCC: Hepatocellular carcinoma
DOX: Doxorubicin
HS: HepaSphere
HPLC: High-performance liquid chromatography
